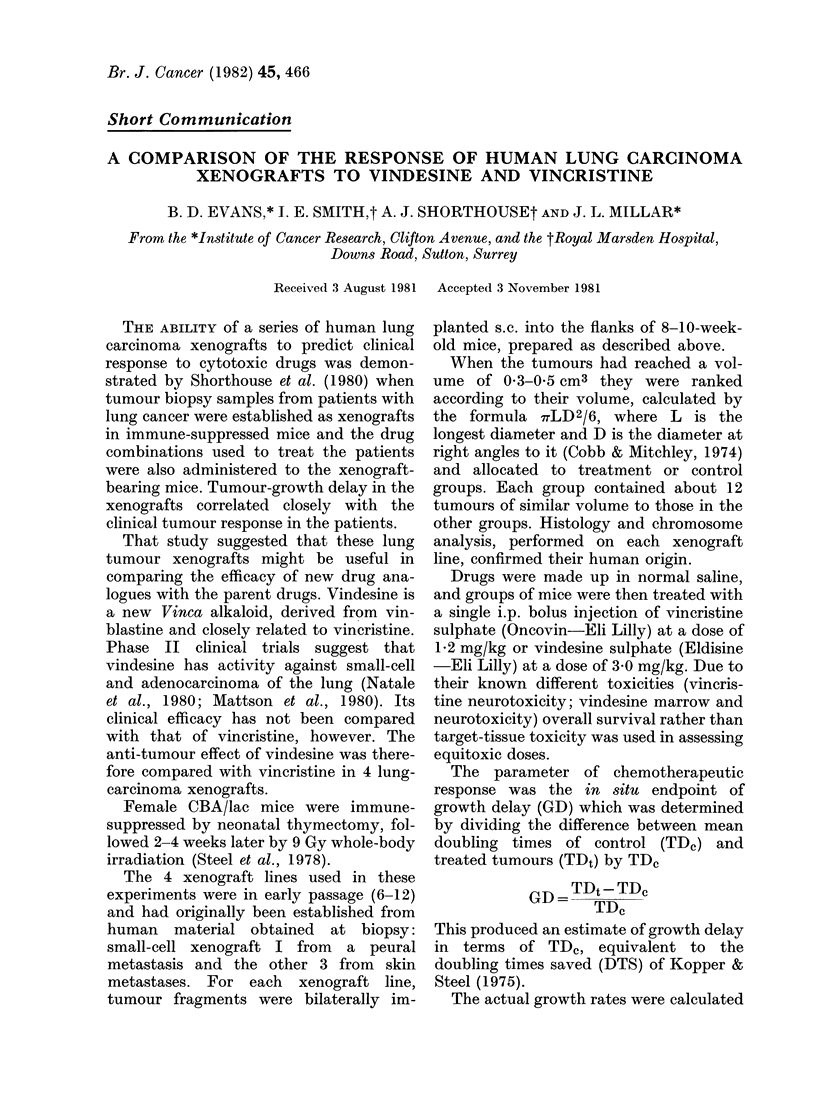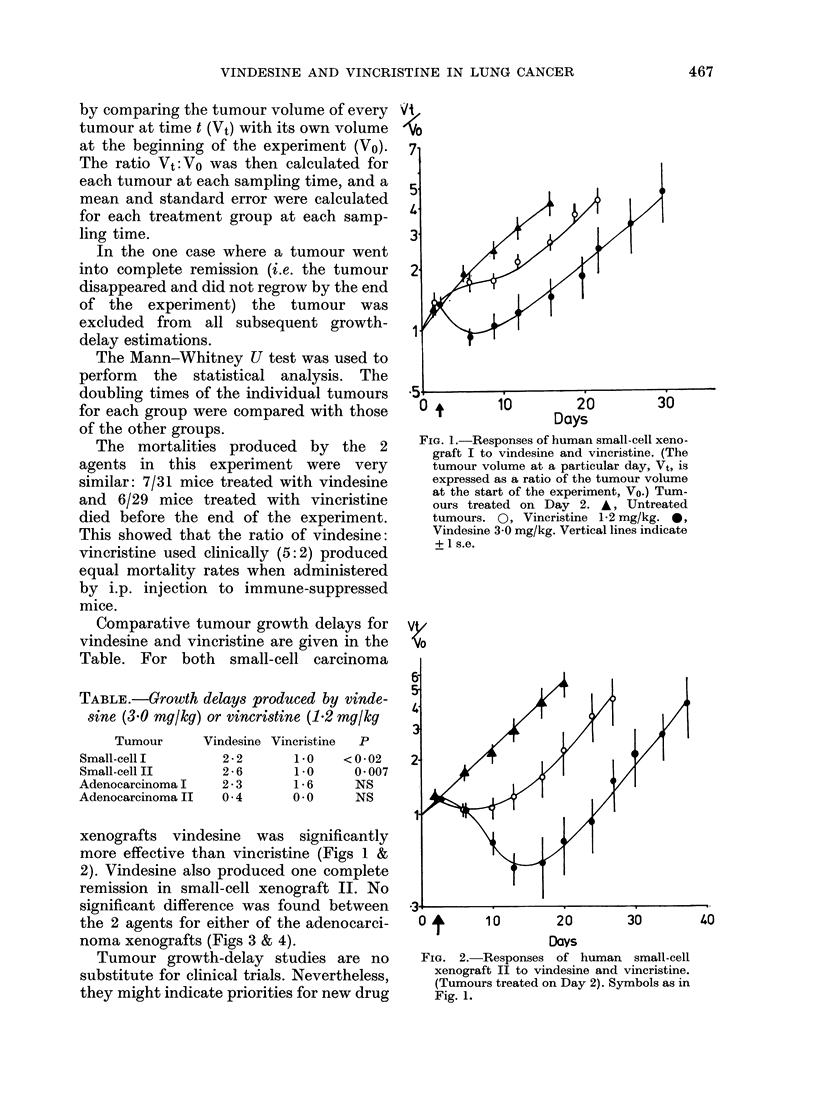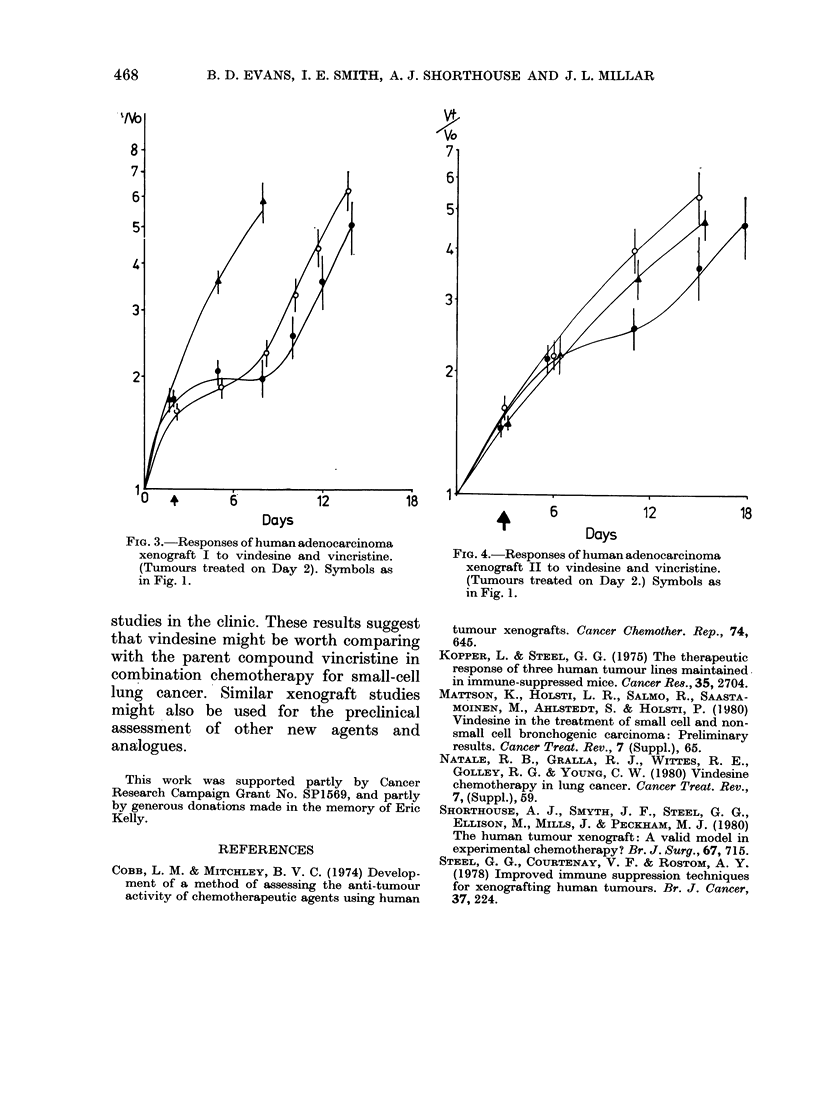# A comparison of the response of human lung carcinoma xenografts to vindesine and vincristine.

**DOI:** 10.1038/bjc.1982.75

**Published:** 1982-03

**Authors:** B. D. Evans, I. E. Smith, A. J. Shorthouse, J. L. Millar


*Br. J. Cancer* (1982) **45**, 466

### *Short Communication*

### A COMPARISON OF THE RESPONSE OF HUMAN LUNG CARCINOMA XENOGRAFTS TO VINDESINE AND VINCRISTINE

B. D. EVANS,* I. E. SMITH,† A. J. SHORTHOUSE† AND J. L. MILLAR*

*From the *Institute of Cancer Research, Clifton Avenue, and the †Royal Marsden Hospital, Downs Road, Sutton, Surrey*

Received 3 August 1981 Accepted 3 November 1981

THE ABILITY of a series of human lung carcinoma xenografts to predict clinical response to cytotoxic drugs was demonstrated by Shorthouse *et al.* (1980) when tumour biopsy samples from patients with lung cancer were established as xenografts in immune-suppressed mice and the drug combinations used to treat the patients were also administered to the xenograft-bearing mice. Tumour-growth delay in the xenografts correlated closely with the clinical tumour response in the patients.

That study suggested that these lung tumour xenografts might be useful in comparing the efficacy of new drug analogues with the parent drugs. Vindesine is a new *Vinca* alkaloid, derived from vinblastine and closely related to vincristine. Phase II clinical trials suggest that vindesine has activity against small-cell and adenocarcinoma of the lung (Natale *et al.*, 1980; Mattson *et al.*, 1980). Its clinical efficacy has not been compared with that of vincristine, however. The anti-tumour effect of vindesine was therefore compared with vincristine in 4 lung-carcinoma xenografts.

Female CBA/lac mice were immune-suppressed by neonatal thymectomy, followed 2–4 weeks later by 9 Gy whole-body irradiation (Steel *et al.*, 1978).

The 4 xenograft lines used in these experiments were in early passage (6–12) and had originally been established from human material obtained at biopsy: small-cell xenograft I from a peural metastasis and the other 3 from skin metastases. For each xenograft line, tumour fragments were bilaterally implanted s.c. into the flanks of 8–10-week-old mice, prepared as described above.

When the tumours had reached a volume of 0·3–0·5 $cm^3$ they were ranked according to their volume, calculated by the formula $\pi LD^2/6$, where L is the longest diameter and D is the diameter at right angles to it (Cobb & Mitchley, 1974) and allocated to treatment or control groups. Each group contained about 12 tumours of similar volume to those in the other groups. Histology and chromosome analysis, performed on each xenograft line, confirmed their human origin.

Drugs were made up in normal saline, and groups of mice were then treated with a single i.p. bolus injection of vincristine sulphate (Oncovin—Eli Lilly) at a dose of 1·2 mg/kg or vindesine sulphate (Eldisine—Eli Lilly) at a dose of 3·0 mg/kg. Due to their known different toxicities (vincristine neurotoxicity; vindesine marrow and neurotoxicity) overall survival rather than target-tissue toxicity was used in assessing equitoxic doses.

The parameter of chemotherapeutic response was the *in situ* endpoint of growth delay (GD) which was determined by dividing the difference between mean doubling times of control ($TD_c$) and treated tumours ($TD_t$) by $TD_c$

$$GD = \frac{TD_t - TD_c}{TD_c}$$

This produced an estimate of growth delay in terms of $TD_c$, equivalent to the doubling times saved (DTS) of Kopper & Steel (1975).

The actual growth rates were calculated by comparing the tumour volume of every tumour at time *t* ($V_t$) with its own volume at the beginning of the experiment ($V_0$). The ratio $V_t{:}V_0$ was then calculated for each tumour at each sampling time, and a mean and standard error were calculated for each treatment group at each sampling time.

In the one case where a tumour went into complete remission (*i.e.* the tumour disappeared and did not regrow by the end of the experiment) the tumour was excluded from all subsequent growth-delay estimations.

The Mann–Whitney *U* test was used to perform the statistical analysis. The doubling times of the individual tumours for each group were compared with those of the other groups.

The mortalities produced by the 2 agents in this experiment were very similar: 7/31 mice treated with vindesine and 6/29 mice treated with vincristine died before the end of the experiment. This showed that the ratio of vindesine: vincristine used clinically (5:2) produced equal mortality rates when administered by i.p. injection to immune-suppressed mice.

Comparative tumour growth delays for vindesine and vincristine are given in the Table. For both small-cell carcinoma xenografts vindesine was significantly more effective than vincristine (Figs 1 & 2). Vindesine also produced one complete remission in small-cell xenograft II. No significant difference was found between the 2 agents for either of the adenocarcinoma xenografts (Figs 3 & 4).

Tumour growth-delay studies are no substitute for clinical trials. Nevertheless, they might indicate priorities for new drug

TABLE.—*Growth delays produced by vindesine (3·0 mg/kg) or vincristine (1·2 mg/kg*

| Tumour | Vindesine | Vincristine | *P* |
|---|---|---|---|
| Small-cell I | 2·2 | 1·0 | <0·02 |
| Small-cell II | 2·6 | 1·0 | 0·007 |
| Adenocarcinoma I | 2·3 | 1·6 | NS |
| Adenocarcinoma II | 0·4 | 0·0 | NS |

FIG. 1.—Responses of human small-cell xenograft I to vindesine and vincristine. (The tumour volume at a particular day, $V_t$, is expressed as a ratio of the tumour volume at the start of the experiment, $V_0$.) Tumours treated on Day 2. ▲, Untreated tumours. ○, Vincristine 1·2 mg/kg. ●, Vindesine 3·0 mg/kg. Vertical lines indicate ±1 s.e.

FIG. 2.—Responses of human small-cell xenograft II to vindesine and vincristine. (Tumours treated on Day 2). Symbols as in Fig. 1.

FIG. 3.—Responses of human adenocarcinoma xenograft I to vindesine and vincristine. (Tumours treated on Day 2). Symbols as in Fig. 1.

FIG. 4.—Responses of human adenocarcinoma xenograft II to vindesine and vincristine. (Tumours treated on Day 2.) Symbols as in Fig. 1.

studies in the clinic. These results suggest that vindesine might be worth comparing with the parent compound vincristine in combination chemotherapy for small-cell lung cancer. Similar xenograft studies might also be used for the preclinical assessment of other new agents and analogues.

This work was supported partly by Cancer Research Campaign Grant No. SP1569, and partly by generous donations made in the memory of Eric Kelly.

### REFERENCES

COBB, L. M. & MITCHLEY, B. V. C. (1974) Development of a method of assessing the anti-tumour activity of chemotherapeutic agents using human tumour xenografts. *Cancer Chemother. Rep.*, **74**, 645.

KOPPER, L. & STEEL, G. G. (1975) The therapeutic response of three human tumour lines maintained in immune-suppressed mice. *Cancer Res.*, **35**, 2704.

MATTSON, K., HOLSTI, L. R., SALMO, R., SAASTAMOINEN, M., AHLSTEDT, S. & HOLSTI, P. (1980) Vindesine in the treatment of small cell and non-small cell bronchogenic carcinoma: Preliminary results. *Cancer Treat. Rev.*, **7** (Suppl.), 65.

NATALE, R. B., GRALLA, R. J., WITTES, R. E., GOLLEY, R. G. & YOUNG, C. W. (1980) Vindesine chemotherapy in lung cancer. *Cancer Treat. Rev.*, **7**, (Suppl.), 59.

SHORTHOUSE, A. J., SMYTH, J. F., STEEL, G. G., ELLISON, M., MILLS, J. & PECKHAM, M. J. (1980) The human tumour xenograft: A valid model in experimental chemotherapy? *Br. J. Surg.*, **67**, 715.

STEEL, G. G., COURTENAY, V. F. & ROSTOM, A. Y. (1978) Improved immune suppression techniques for xenografting human tumours. *Br. J. Cancer*, **37**, 224.